# Brucella-Induced Ruptured Infrarenal Dissecting Abdominal Aortic Aneurysm

**DOI:** 10.1055/s-0039-1688449

**Published:** 2019-09-17

**Authors:** Harishankar Ramachandran Nair, Prakash Goura, Shivanesan Pitchai, Unnikrishnan Madathipat

**Affiliations:** 1 Department of Vascular and Thoracic Surgery, Sree Chitra Tirunal Institute for Medical Sciences and Technology, Trivandrum, Kerala, India

**Keywords:** ruptured mycotic aortic aneurysm, open aneurysm repair, Brucella melitensis

## Abstract

Mycotic aneurysms, often saccular, accounting for approximately 2.5% of all abdominal aortic aneurysms, possess increased risk of rupture, uncontrolled sepsis, and protracted hospital stay and are associated with high morbidity and mortality. The authors report the case of a 49-year-old female with no known comorbidities who presented with free rupture of an infrarenal dissecting mycotic aneurysm and underwent emergent open repair successfully. The etiological agent, *Brucella melitensis* , a Gram-negative zoonotic coccobacillus, is rarely reported to cause mycotic aneurysm.

## Introduction

Free rupture of infrarenal abdominal aortic aneurysm is a catastrophic event leading to imminent mortality, unless expeditiously intervened. Infective aneurysms have higher notoriety in terms of associated septicemia and related complications. Although endovascular aneurysm repair has currently come to center stage for management of even ruptured aneurysms, when suspecting an infected lesion, the therapeutic option of choice is open surgical repair, particularly in younger patients.

## Case Presentation

**Fig. 1:**
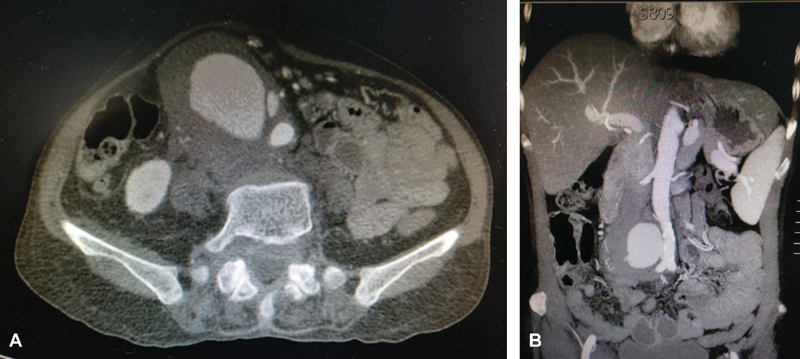
Computed tomography angiogram (axial [Panel A] and coronal [Panel B] views) showing saccular infrarenal aortic aneurysm with significant eccentric thrombus and focal dissection above aortic bifurcation.

A 49-year-old housewife weighing 45 kg, with no known comorbidities, presented with malaise, loss of appetite, weight loss, recurrent low-grade fever for 1 month, and intractable lower abdominal pain for 2 weeks. Clinically, she was frail and thin, but hemodynamically stable. Abdominal examination revealed a tender, wide pulsatile mass in the periumbilical and right lumbar regions. Abdominal computed tomography showed an 8-cm infrarenal saccular aneurysm ( Fig. 1 ). Blood culture was sent because suspicion of mycotic aneurysm was high.

**Fig. 2:**
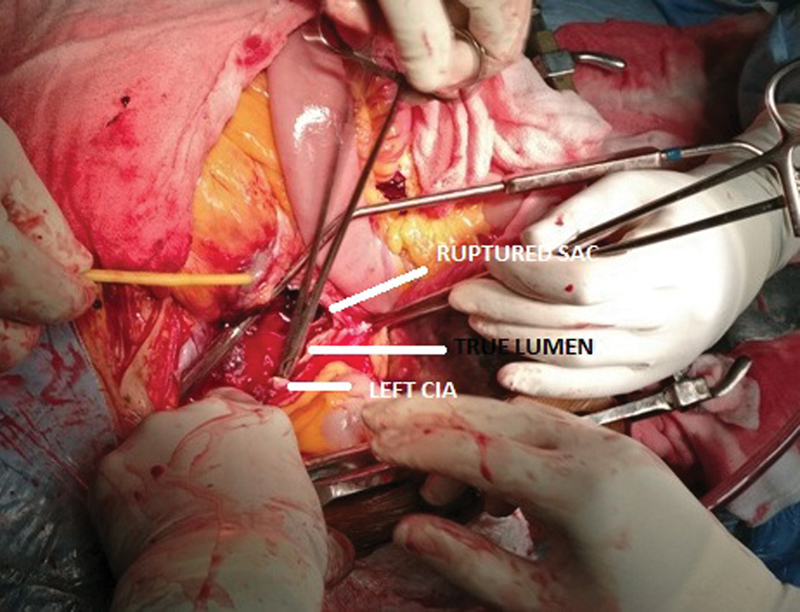
Intraoperative pictures showing true lumen (right angled clamp inside) and ruptured false lumen/sac; Foley's is being used for occluding common iliac arteries (CIAs).

One hour after admission, she suddenly collapsed. Cardiopulmonary resuscitation was initiated and she was transferred to the intensive care unit. Her abdomen was distended and the patient was pale, suggesting free rupture of the aneurysm. She was rushed to the operating room. Quick thoracotomy was made through the left sixth intercostal space for proximal aortic control, followed by midline laparotomy. There was 2 L of frank blood and 1 L of fresh clots in the peritoneal cavity. A dissecting saccular aneurysm was found originating approximately 2 cm above the iliac bifurcation which had ruptured into the base of the small bowel mesentery and thereafter into the peritoneal cavity ( Fig. 2 ). Both common iliac arteries were friable. She underwent aortobifemoral bypass with a 14 × 7 mm collagen-coated knitted polyester graft (MAQUET INTERGARD K), along with suture closure of both common iliac arteries. Her postoperative and subsequent course in hospital was uneventful. Blood culture and clot from the aneurysm grew the Gram-negative coccobacillus *Brucella melitensis.* She was started on culture-specific antibiotics and was discharged in stable condition on postoperative day 9, on long-term oral antibiotics (rifampicin and doxycycline).

## Discussion

The term “mycotic” was coined by Sir William Osler 1 in 1885. This is actually a misnomer now, since not all infective aneurysms are caused by fungi. At that time, the term “mycotic” was applied to any infection of bacterial or fungal origin. So, Jarrett et al 2 applied the term “infected” for such aneurysms in their case series. The pathophysiology is not clearly known but is usually secondary to endocarditis, contiguous aortitis, direct inoculation (intravenous drug abuse, trauma, or arterial catheterization), or microembolization of vasa vasorum. The commonly affected segment of the aorta is the infrarenal (66%) followed by the ascending/thoracic (25%). The infectious etiology is commonly bacterial in origin due to translocation from gut in severely immunocompromised patients or secondary to septicemia. Common agents implicated are salmonella, staphylococcus, *Escherichia coli* , tuberculosis, fungi, and rarely syphilis.

However, the pathogen isolated in our patient was *Brucella melitensis* , 3 4 shown by positive blood culture, culture of clot from aneurysm, and high Brucella immunoglobulin M antibodies. It is a zoonotic intracellular Gram-negative coccobacillus which causes multisystemic Malta fever, undulant fever, respiratory infections, and septicemia, especially in immunocompromised patients. It is a very rare cause for primary mycotic aortic aneurysm, 3 5 6 although there are a few case reports of primary endocarditis, ensuing septicemia, and associated focal aneurysms 4 7 of the aorta or other arteries. The mode of spread is usually from infected cattle directly, or more commonly via milk and other body fluids. Our patient most probably contracted the pathogen from infected milk or milk products. Brucella species is known to produce outbreaks in cattle and zoonosis in nearby human populations.

Mycotic aneurysms have a high propensity for complications like rupture, aortoenteric fistula, and graft-related complications and often have a protracted postoperative course. Mortality 6 7 is as high as 40% in some case series. General principles of surgical treatment 7 8 include obtaining tissue specimen for Gram stain and tissue culture, wide debridement of all infected tissues including infected arterial wall, copious irrigation of the surgical field with antiseptic solution, and arterial reconstruction followed by prolonged postoperative use of specific antibiotics.

Prodromal infective features followed by ruptured aortic aneurysm in a young patient with atypical clinical features and no specific risk factors can be due to rare organisms (like Brucella in our case).This situation mandates emergent intervention and prolonged antibiotic therapy. Periodic screening for possible recurrence and prosthetic graft infection is mandatory.
